# Dexmedetomidine promotes apoptosis and suppresses proliferation of hepatocellular carcinoma cells via microRNA-130a/EGR1 axis

**DOI:** 10.1038/s41420-021-00805-5

**Published:** 2022-01-19

**Authors:** Lei Zhou, Juanni Li, Xing Liu, Yongzhong Tang, Tunliang Li, Huiyin Deng, Jia Chen, Xinlin Yin, Kuan Hu, Wen Ouyang

**Affiliations:** 1 Department of Anesthesiology, The Third Xiangya Hospital, Central South University, 410013 Changsha, Hunan China; 2 Department of Pathology, Xiangya Hospital, Central South University, 410008 Changsha, Hunan China; 3 Key Laboratory of Medical Information Research, College of Hunan Province, Central South University, Changsha, China; 4 Department of Hepatobiliary Surgery, Xiangya Hospital, Central South University, 410008 Changsha, Hunan China; 5 The Seniors Anesthesia and Perioperative Management Research Center, Central South University, Changsha, Hunan China; 6 The State Key Laboratory of Medical Genetics, School of Life Sciences, Central South University, Changsha, Hunan China

**Keywords:** Cell biology, Diseases

## Abstract

Accumulating evidence has revealed the role of microRNAs (miRs) in hepatocellular carcinoma (HCC). Dexmedetomidine, a highly selective α_2_-adrenergic agonist, is widely used in perioperative settings for analgesia and sedation. Herein, we aimed to determine whether dexmedetomidine might directly regulate miR-130a/early growth response 1 (EGR1) axis in HCC and explore the related mechanisms. miR-130a and EGR1 expression were determined in HCC tissues and their correlation was evaluated. Human HCC cell line HCCLM3 was selected. Upon the determination of the optimal concentration of dexmedetomidine, HCCLM3 cells were treated with dexmedetomidine, miR-130a- or EGR1-related oligonucleotides or plasmids were transfected into cells to explore their functions in cell biological behaviors. miR-130a and EGR1 levels in cells were tested. The targeting relationship between miR-130a and EGR1 was verified. miR-130a was inhibited while EGR1 was elevated in HCC tissues and they were negatively correlated. EGR1 was targeted by miR-130a. With the increase of dexmedetomidine concentration, HCCLM3 cell viability was correspondingly inhibited, miR-130a expression was elevated and EGR1 expression was decreased. Dexmedetomidine, upregulating miR-130a or downregulating EGR1 inhibited proliferation, invasion and migration, and promoted apoptosis of HCCLM3 cells. MiR-130a upregulation/downregulation enhanced/impaired the effect of dexmedetomidine on cell biological behaviors. Our study provides evidence that raising miR-130a enhances the inhibitory effects of dexmedetomidine on HCC cellular growth via inhibiting EGR1. Thus, miR-130a may be a potential candidate for the treatment of HCC.

## Introduction

Hepatocellular carcinoma (HCC) is the commonest primary malignant tumor of hepatocytes, the fifth frequent cancer and the third leading cause of cancer-related mortality globally, after lung cancer and stomach cancer [1]. HCC may be frequently diagnosed and it can be possible exclusively by cross-sectional imaging on the basis of characteristic multiphase contrast [2]. HCC most commonly occurs with chronic alcohol abuse, hepatitis C virus (HCV), or nonalcoholic fatty liver disease [3]. Locoregional therapy, hepatic resection, and liver transplantation may be effective in the early stages of the tumor which account for less than 30% of patients, while transarterial chemoembolization is the first-line treatment for advanced HCC [4]. Metastasis is the major cause of the high mortality of HCC patients post-surgical resection [5]. And even after liver resection, the risk of tumor recurrence may exceed 70% within 5 years [6]. Therefore, it is urgent to develop novel biomarkers in the early prognosis and assessment of treatments for HCC.

Dexmedetomidine (DEX) is a potent, highly, selective α_2_-adrenergic agonist accompanied with intrinsic analgesic properties and anxiolytic, sedative, and sympatholytic effects [7]. It has been revealed that a satisfactory anesthetic effect could be obtained in HCC surgery with DEX combined with propofol via percutaneous microwave coagulation therapy [8]. It is reported that DEX reduces cell apoptosis and lipopolysaccharide-induced liver oxidative stress in rats via the α_2_ adrenergic receptor [9]. MicroRNAs (miRNAs) are non-protein coding RNA molecule > 18–22 nt, which are closely related to the modulation of cell phenotypes, including cell proliferation, apoptosis, and differentiation [10]. A study has revealed that miR-130a is declined in HCC while its overexpressed gene targets are primarily related to aberrant cell proliferation which participates in nucleotide metabolism, DNA replication, and transcription [11]. Another study has demonstrated that the hepatitis B virus-related estrogen receptor alpha is modulated by miR-130a in HepG2.2.15 human HCC cells [12]. Early growth response 1 (EGR1) pertains to the EGR family of C2H2-type zinc-finger proteins, which is a transcriptional modulator and controls many target genes participated in cell proliferation, differentiation, and survival [13]. A study has suggested that EGR1 plays an antitumor role by downregulating the transcriptional level of CD24A, a functional liver tumor-initiating cell marker, in HCC [14]. Another study has revealed that EGR1 enhances hypoxia-induced autophagy to raise chemo-resistance of HCC cells [15]. At present, there is no research on the target relationship between miR-130a and EGR1 in HCC. Thus, in this study, the function and mechanism of DEX and miR-130a/EGR1 axis in HCC cells were investigated.

## Results

### miR-130a is downregulated and EGR1 is upregulated in HCC; miR-130a targets EGR1

**Fig. 1:**
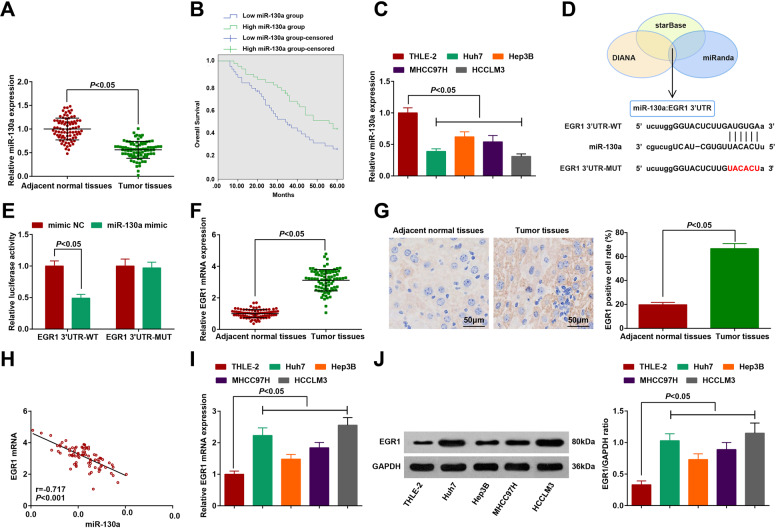
HCC tissues and cells show reduced miR-130a and elevated EGR1; miR-130a targets EGR1. **A** miR-130a expression in HCC tissues and adjacent normal tissues detected by RT-qPCR (*n* = 83); **B** The prognostic value of miR-130a in HCC patients; **C** miR-130a expression in HCC cells detected by RT-qPCR (*N* = 3); **D** Predicted binding sites of miR-130a and EGR1 3’-UTR, and mutant sites of EGR1 3’-UTR in EGR-WT reporter; **E** The targeting relation between miR-130a and EGR1 verified by dual-luciferase reporter gene assay; **F** EGR1 expression in HCC tissues and adjacent normal tissues detected by RT-qPCR (*n* = 83); **G** EGR1 expression in HCC tissues and adjacent normal tissues detected by immunohistochemistry (scale bar = 50 μm; *n* = 83); **H** Correlation between EGR1 and miR-130a expression in HCC tissues detected by Pearson test (*n* = 83); **I, J** EGR1 expression in normal hepatocytes and HCC cells detected by RT-qPCR and western blot analysis (*N* = 3); The data were expressed as mean ± standard deviation and compared by *t*-test or one-way ANOVA.

First, miR-130a in HCC tissues and adjacent normal tissues was tested by RT-qPCR. In HCC tissues, miR-130a expression was downregulated (Fig. 1A). The relationship between miR-130a expression and clinicopathological characteristics of HCC patients was evaluated. All patients were divided into miR-130a high expression group and miR-130a low expression group using the median level of miR-130a as the cutoff value. The findings implied that miR-130a expression levels were correlated with tumor size (*P* = 0.014), TNM stage (*P* = 0.019), and tumor differentiation (*P* = 0.024) (Table 1). Additionally, patients with low expression of miR-130a had poorer overall survival (Fig. 1B). A summary table (Table 2) detailed the expression of miR-130a in tumor and adjacent normal tissues. miR-130a expression was also detected in HCC cell lines. It was manifested that miR-130a was downregulated in HCC cell lines (Huh7, Hep3B, MHCC97H, and HCCLM3) (Fig. 1C). HCCLM3 cells were selected for subsequent experiments

**Table 1:** The relationship between miR-130a expression and clinicopathological characteristics of patients with HCC.

Variables	Cases (*n* = 83)	miR-130a expression		*P*-value
		High (*n* = 42)	Low (*n* = 41)	
Age (years)				0.658
≤55	35	19	16	
>55	48	23	25	
Gender				0.314
Female	20	8	12	
Male	63	34	29	
HBV infection				0.469
Present	59	28	31	
Absent	24	14	10	
AFP (ng/mL)				0.119
≤200	34	21	13	
>200	49	21	28	
Tumor size (cm)				0.014
≤5	50	31	19	
>5	33	11	22	
Cellular differentiation				0.024
I–II	62	36	26	
III–IV	21	6	15	
TNM tumor stage				0.019
I–II	57	34	23	
III–IV	26	8	18	

*HBV* Hepatitis B Virus, *AFP* alpha-fetoprotein, *TNM* Tumor node metastasis.

**Table 2:** Expression of miR-130a in tumor and adjacent normal tissues.

Markers	Adjacent normal tissues	Tumor tissues
miR-130a	High	Low
EGR1	Low	High

The target prediction tools, including miRanda, starBase, and DIANA, were applied to identify potential targets of miR-130a. Then, EGR1 (Fig. 1D) was picked because of its relation to HCC progression [16–18]. To further test whether EGR1 was a direct target of miR-130a, EGR1 3-‘UTR-WT, and 3’UTR-MUT were cloned to psi-CHECK2. Then, the results demonstrated that miR-130a mimic reduced the luciferase activity of EGR1 3’-UTR-WT in HCCLM3 cells (Fig. 1E) but not affect that of EGR1 3’-UTR-MUT.

EGR1 expression in HCC tissues and adjacent normal tissues was tested by RT-qPCR and immunohistochemistry. In HCC tissues, EGR1 expression was upregulated (Fig. 1F, G). Moreover, The Pearson correlation analysis revealed a remarkably negative correlation between the expression of miR-130a and EGR1 in HCC tissues (r = −0.717, *P* < 0.001, Fig. 1H). EGR1 expression was also detected in HCC cell lines. It was manifested that EGR1 was upregulated in HCC cell lines (Huh7, Hep3B, MHCC97H, and HCCLM3) (Fig. 1I, J). The results confirmed that the dysregulation of miR-130a/EGR1 axis may be involved in the occurrence and development of HCC.

### DEX suppresses the viability of HCCLM3 cells

**Fig. 2:**
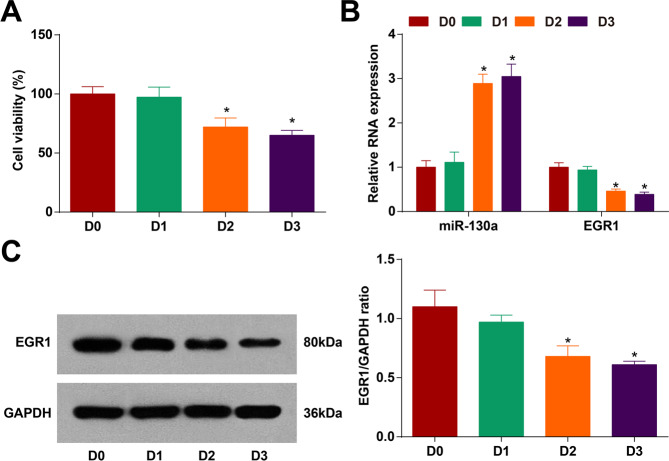
HCC cell proliferation is suppressed by DEX. **A** Detection of cell viability by trypan blue staining; **B, C** miR-130a and EGR1 expression in HCC cells treated with DEX of different concentrations detected by RT-qPCR and western blot analysis (D0: DEX with a concentration of 0; D1: DEX with a concentration of 1 nmol/L; D2: DEX with a concentration of 10 nmol/L; D3: DEX with a concentration of 100 nmol/L). *N* = 3. The data were expressed as mean±standard deviation and compared by one-way ANOVA. *p < 0.05 vs. the D0 group.

Trypan blue staining revealed that (Fig. 2A) DEX of 1 nmol/L had no effect on the viability of HCCLM3 cells, while DEX of 10 nmol/L and 100 nmol/L showed a suppressive effect on cell viability. There was no marked difference between the two concentrations, thus 10 nmol/L DEX was selected for the follow-up experiments.

RT-qPCR and western blot analysis detected miR-130a and EGR1 expression in cells treated with different concentrations of DEX. It was displayed that with the increase of DEX concentration (from 10 nmol/L), HCCLM3 cell viability was inhibited accordingly, miR-130a expression was elevated and EGR1 expression was reduced (Fig. 2B, C).

### DEX or upregulated miR-130a suppresses the growth of HCC cells

**Fig. 3:**
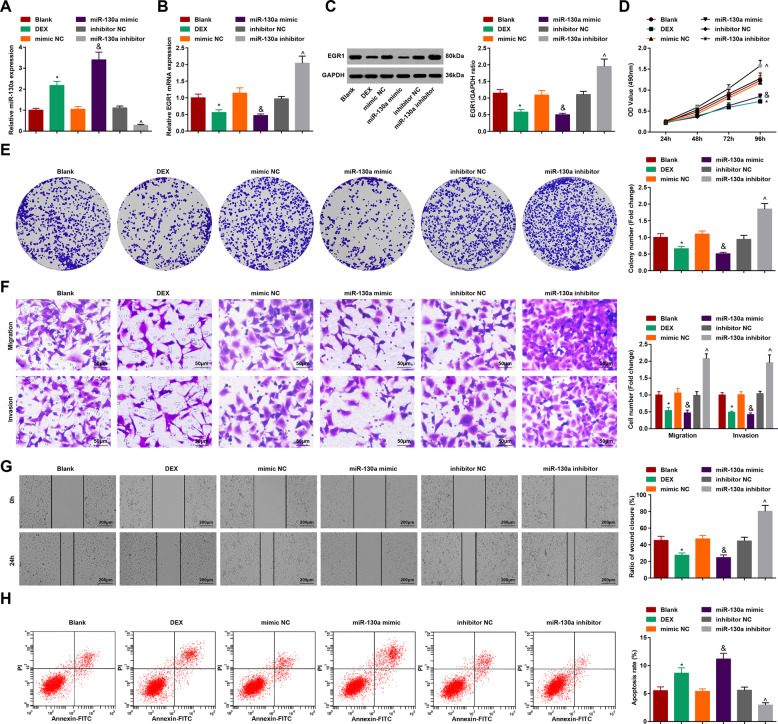
DEX or upregulated miR-130a suppresses the growth of HCC cells. **A** miR-130a expression tested by RT-qPCR after DEX treatment or upregulating miR-130a; **B, C** Detection of EGR1 expression in cells tested by RT-qPCR and western blot analysis after DEX treatment or upregulating miR-130a; **D** OD value tested by MTT test after DEX treatment or upregulating miR-130a; **E** Colony-forming ability of HCC cells tested by colony formation assay after DEX treatment or upregulating miR-130a; **F** Migration and invasion of HCC cells tested by Transwell test after DEX treatment or upregulating miR-130a (scale bar = 50 μm); **G** Migration distance of HCC cells tested by scratch test after DEX treatment or upregulating miR-130a (scale bar = 200 μm); **H** Apoptosis of HCC cells tested by flow cytometry after DEX treatment or upregulating miR-130a. *N* = 3. The data were expressed as mean±standard deviation and compared by one-way ANOVA. *p < 0.05 vs. the blank group; ^&^*p* < 0.05 *vs*. the mimic NC group; ^^^*p* < 0.05 vs. the inhibitor NC group.

RT-qPCR showed that miR-130a level was increased in HCC cells transfected with miR-130a mimic while decreased in cells transfected with miR-130a inhibitor (Fig. 3A). western blot analysis indicated that (Fig. 3B, C) either DEX or elevated miR-130a could reduce EGR1 expression while reduction of miR-130a could elevate EGR1 expression.

Next, MTT assay and colony formation assay, scratch test, Transwell assay, and flow cytometry (Fig. 3D-H) revealed that DEX or miR-130a upregulation suppressed the proliferation, colony formation, migration, and invasion, and elevated apoptosis of HCCLM3 cells. miR-130a downregulation functioned oppositely on HCCLM3 cells.

Therefore, a conclusion was drawn that DEX or upregulated miR-130a suppressed the growth of HCC cells.

### Silencing EGR1 inhibits HCC progression

**Fig. 4:**
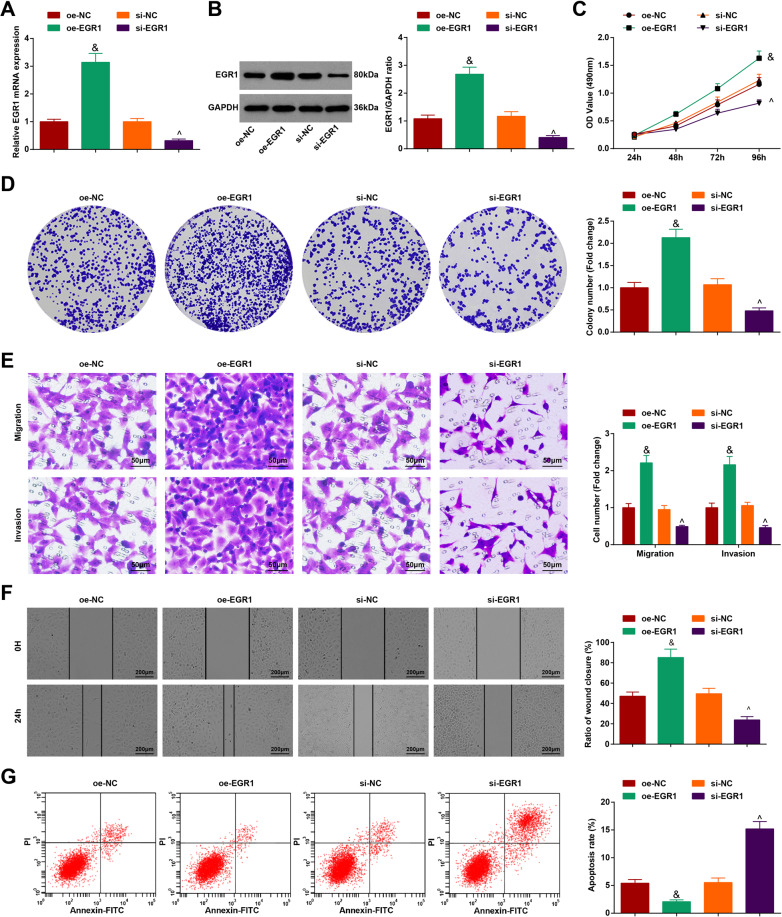
Silencing EGR1 inhibits HCC progression. **A, B** EGR1 expression in HCC cells tested by RT-qPCR and western blot analysis after upregulating or downregulating EGR1; **C** OD value tested by MTT test after upregulating or downregulating EGR1; **D** Colony-forming ability of HCC cells tested by colony formation assay after upregulating or downregulating EGR1; **E** Migration and invasion of HCC cells tested by Transwell test after upregulating or downregulating EGR1 (scale bar = 50 μm); **F** Migration distance of HCC cells tested by scratch test after upregulating or downregulating EGR1 (scale bar = 200 μm); **G** Apoptosis of HCC cells tested by flow cytometry after upregulating or downregulating EGR1. *N* = 3. The data were expressed as mean±standard deviation and compared by one-way ANOVA. ^&^*p* < 0.05 vs. the oe-NC group; ^^^*p* < 0.05 vs. the si-NC group.

EGR1 is a transcription factor involved in the regulation of cell proliferation and apoptosis, and it is evident that EGR1 promotes the development of prostate cancer [19]. To explore EGR1’s effects in HCC, HCC cells were transfected with oe-EGR1 and si-EGR1. RT-qPCR and western blot analysis detection displayed that (Fig. 4A, B) oe-EGR1 elevated but si-EGR1 reduced EGR1 expression level in HCC cells.

Then, cellular experiments further explored that in HCC cells overexpressing EGR1, the malignant phenotypes were promoted while in those depleting EGR1, cell growth was inhibited (Fig. 4C-G).

The outcomes indicated that depletion of EGR1 inhibited the biological activities of HCC cells.

### Elevating miR-130a enhances the inhibitory effects of DEX on HCC

**Fig. 5:**
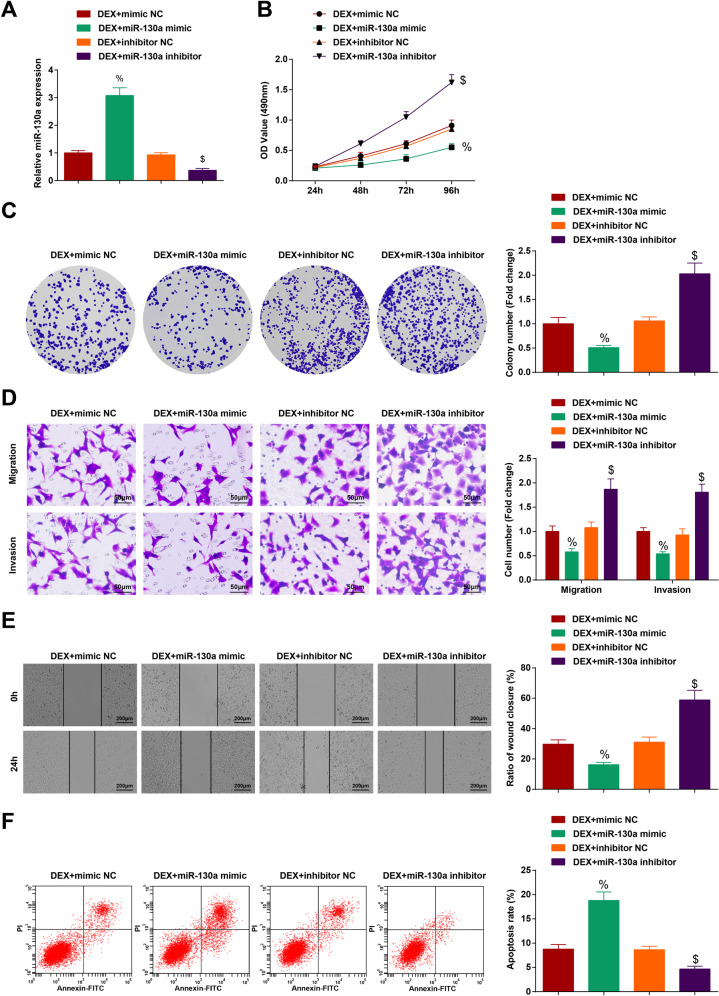
Elevating miR-130a enhances the inhibitory effects of DEX on HCC. **A** miR-130a expression tested by RT-qPCR after DEX treatment and upregulating miR-130a; **B** OD value tested by MTT test after DEX treatment and upregulating miR-130a; **C** Colony-forming ability of HCC cells tested by colony formation assay after DEX treatment and upregulating miR-130a; **D** Migration and invasion of HCC cells tested by Transwell test after DEX treatment and upregulating miR-130a (scale bar = 50 μm); **E** Migration distance of HCC cells tested by scratch test after DEX treatment and upregulating miR-130a (scale bar = 200 μm); **F** Apoptosis of HCC cells tested by flow cytometry after DEX treatment and upregulating miR-130a. *N* = 3. The data were expressed as mean±standard deviation and compared by one-way ANOVA. ^%^*p* < 0.05 vs. the DEX + mimic NC group; ^$^*p* < 0.05 vs. the DEX + inhibitor NC group.

To further clarify the relationship between DEX, miR-130a, and HCC, we treated HCC cells with DEX and transfected with miR-130a mimic/inhibitor. Then, it was measured by RT-qPCR that transfection of miR-130a mimic elevated but that of miR-130a inhibitor reduced miR-130a expression in DEX-treated HCC cells (Fig. 5A). Also, it was observed that elevating miR-130a further suppressed the malignant progression of DEX-treated HCC cells. On the contrary, knocking down miR-130a had the opposite functions on DEX-treated HCC cells (Fig. 5B-F). It was informed that DEX mediated miR-130a/EGR1 axis in HCC process.

## Discussion

**Fig. 6:**
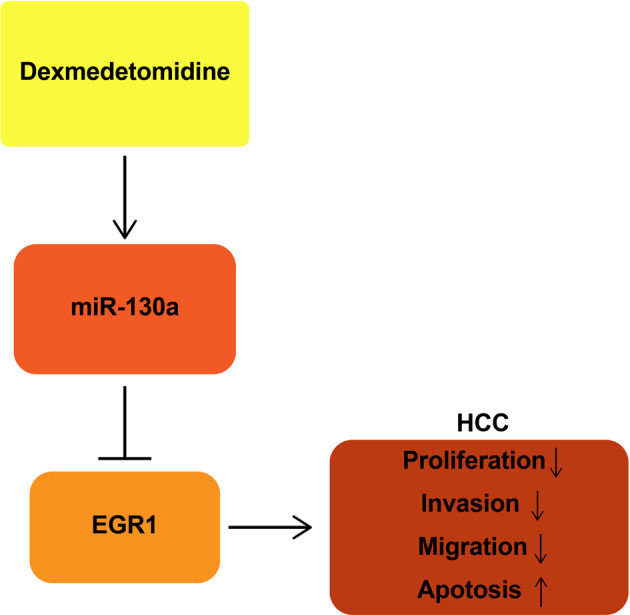
The mechanistic diagram. Dex upregulates miR-130a to suppress EGR1 expression, thereby inhibiting the proliferation, invasion and migration of and promoting apoptosis of HCC cells.

HCC is a highly invasive malignant tumor with a poor prognosis [20]. In this work, we have identified that DEX or miR-130a overexpression can inhibit the proliferation, invasion and migration, promoted apoptosis of HCC cells while miR-130a inhibition functions with the opposite effects. In addition, we discovered that miR-130a upregulation enhanced DEX-induced inhibitory effects on HCC cell progression while miR-130a inhibition reversed those effects on HCC. Shortly, we have delineated that DEX upregulated miR-130a to suppress EGR1 expression, thereby impeding HCC cell progression (Fig. 6).

Unlike opioids and other commonly used sedatives (such as propofol, fentanyl, and midazolam), DEX works out sedative effects without causing respiratory depression [21]. DEX allows patients to breathe spontaneously during sedation, thereby reducing the risk of respiratory depression [22]. Except for the sedative effects, DEX also exhibits antitumor effects just like morphine [23], propofol [24], midazolam [25], diazepam [26], sufentanil [27], tramadol [28], and many other classic sedative and analgesic drugs. To explore the performance of DEX in HCC, we performed in vitro experiments, and eventually unveiled that DEX suppressed HCC cell development. A study has shown that DEX blocks cell proliferation, migration, and invasion and accelerates cell apoptosis in ovarian cancer [29], while another study has implied that DEX inhibits osteosarcoma cell proliferation and migration, and promotes apoptosis by regulating miR-520a-3p [30]. As for the role of DEX in HCC, a study has indicated that cell apoptosis in liver tissues is reduced after DEX treatment [31]. Another study has revealed that DEX downregulates cleavage of caspase-3, thus suppressing apoptosis of hepatocytes [32]. Furthermore, evidence has shown that DEX could attenuate the proliferation of liver cells [33]. However, it is previously discovered that DEX accelerates metastasis of breast, lung, and colon cancers [34]. Adversely, DEX is able to stimulate tumour metastasis after lung cancer surgery [35]. Therefore, the promoting or inhibitory functions of DEX in tumor progression vary in different situations.

Emerging evidence has shown that miRNAs play an essential role in tumorigenesis, acting as oncogenes or tumor suppressors [36]. miR-130a is regarded as a tumor suppressor gene in many human tumors, and its overexpression can effectively inhibit the proliferation and invasion of tumor cells and induce apoptosis [37–39]. To identify whether miR-130a owns the antitumor effect in HCC, we implemented various experiments and finally confirmed that miR-130a overexpression hindered HCC progression. Echoed with our finding [40], miR-130a expression is recently reported to decline, which is correlated with the poor prognosis of HCC patients [41, 42]. In fact, a study has shown that miR-130a attenuates HCV replication through upregulating the innate immune response when HCV infection is the main cause of chronic hepatitis and HCC [43]. It has been suggested that restoration of miR-130a-3p attenuates the cell migration and invasiveness in gemcitabine-resistant HCC cells [40]. It is reported that overexpressed miR-130a resists cisplatin-induced suppression of HCC cell proliferation [44]. Collectively, miR-130a functioned to depress the progression of HCC.

miRNA regulates the target genes in tumors [45]. In this study, we clarified that miR-130a targeted EGR1, which enhanced the aggressiveness of HCC cells. It is customarily considered that EGR1 activates hepatocyte growth factor (HGF)-induced cell invasion in HCC cells and heparin suppresses HGF-induced cellular invasion through the depletion of EGR1 [46]. Also, it is reported that EGR1 expression is markedly heightened in HCC tissues and repressing EGR1 partially suppresses the proliferation of HCC cells [47, 48]. EGR1 overexpression restores the anti-proliferative and migratory properties of HCC cells while EGR1 downregulation owns the opposite functions [49]. There is a study implying that EGR1 induces lncRNA FOXD2-AS1 to upregulate, thereby accelerating HCC progression [50]. Moreover, suppressing EGR1 is documented to impair malignant phenotypes of HCC [51]. Anyway, downregulating EGR1 could delay HCC progression which is consistent with previous researches.

## Conclusion

In conclusion, our findings identify that DEX can suppress the proliferation and facilitate apoptosis of HCC cells via upregulating miR-130a and inhibiting EGR1 expression, thus offering a new target for the treatment of HCC. However, the trial size in the designed experiment was relatively small, therefore, the collected results need further verification in a larger cohort. In the future study, the relative pathways and downstream pathways involved in miR-130a/EGR1 axis regulating HCC shall be extensively explored. Starting from other studies which have been demonstrated that EGR1 suppresses the PI3K/AKT pathway, in the present study the hypothesis is that the axis DEX/miR-130a/EGR1 may also regulate the PI3K/AKT signaling pathway, participating in the proliferation and apoptosis of HCC cells. The hypothesis needs further studies in the future.

## Methods

### Compliance with ethical standards

All animal experiments were in tally with the Guide for the Care and Use of Laboratory Animal of the National Institutes of Health. The protocol was allowed by the Committee on the Ethics of Animal Experiments of The Third Xiangya Hospital, Central South University. All patients signed an informed consent form. The clinical sample collection program was approved by the ethics committee of the Third Xiangya Hospital, and the approval number is “KY2019–125”.

### Specimen collection

Clinical HCC tissue specimens (*n* = 83) and adjacent normal tissues (≥3 cm from the cancer tissues) were resected by general surgery from HCC patients (63 males and 20 females) who were confirmed by pathology in the The Third Xiangya Hospital, Central South University. The specimens were divided into 2 parts: 1 part was immediately placed in liquid nitrogen for later use; the other part was fixed with 4% formaldehyde and paraffin-embedded for routine histological staining.

### Cell culture

Human HCC cell lines Huh7, Hep3B, MHCC97H, and HCCLM3 and normal liver THLE-2 cells (all from Mingzhoubio, Ningbo, China) were cultivated in a 5% CO_2_ incubator with dulbecco’s Modified Eagle Medium (DMEM) containing 10% fetal bovine serum (FBS) (Gibco, Carlsbad, California, USA), 50 μg gentamicin and 50 U/mL penicillin (HyClone Company, Logan, UT, USA). Cells in the logarithmic growth phase were taken for reverse transcription quantitative polymerase chain reaction (RT-qPCR) and western blot detection of miR-130a and EGR1 expression.

### RT-qPCR

The total RNA was extracted from tissues and cells by Trizol kit (Invitrogen). RNA concentration was determined by an ultraviolet spectrophotometer, and RNA was reversely transcribed into cDNA with reference to miScript Reverse Transcription Kit (Takara, Shiga, Japan). According to the instructions of SYBR Premix Ex Taq^TM^ PCR Kit (Takara), the fluorescence quantitative PCR reaction was carried out on the fluorescence quantitative PCR instrument (Roche Diagnostics GmbH, Mannheim, Germany). U6 was the loading control of miR-130a, while GAPDH was that of EGR1. The primer sequences were compounded by Shanghai Genechem Co., Ltd. (Shanghai, China) (Table 3). The data were computed by 2^-ΔΔCt^ method.

**Table 3:** Primer sequence for RT-qPCR.

Genes	Primer sequences
miR-130a	F: 5’–CAGTGCAATGTTAAAAGGGCAT–3’
U6	F: 5’–GCTTCGGCAGCACATATACTAAAAT–3’
EGR1	F: 5’–CGGCAGAAGGACAAGAAAGCAGAC–3’
	R: 5’–GGGGAAGTGGGCAGAAAGGATTG–3’
GAPDH	F: 5’–TGAAGGTCGGAGTCAACGGATTTGGT–3’
	R: 5’–CATGTGGGCCATGAGGTCCACCAC–3’

*F* forward, *R* reverse, *miR-130a* microRNA-130a, *EGR1* early growth response 1, *GAPDH* glyceraldehyde-3-phosphate dehydrogenase.

### Western blot analysis

Tissue and cell protein were extracted with a radio-immunoprecipitation assaylysis buffer, and the protein concentration was detected by the bicinchoninic acid method. Upon sodium dodecyl sulfate polyacrylamide gel electropheresis separation, the protein was transferred onto a polyvinylidene fluoride membrane by a water bath electroporator. The membrane was blocked with 5% skimmed milk, probed with primary antibody against EGR1 (1: 1000), and GAPDH (1: 1000, both from Abcam, MA, USA), and reprobed with secondary antibody labeled with horseradish peroxidase (1: 10,000, Abcam). The image was exposed and scanned. Image J software was adopted for assessing gray value of the target band.

### Immunohistochemistry

The paraffin sections of tumor tissues were hydrated with different concentrations of ethanol (100, 95, 85, 70%) and deionized water, then soaked in citric acid buffer (0.01 mol/L, pH 6.0), and heated at 95–100 °C. Then, the sections were added with 0.5% Triton × 100, stained with biotin-streptavidin HRP (ZSGB, China) and incubated with the corresponding antibody overnight. The brown stain on the membrane indicated a positive immunoreaction. The percentage of positively stained cells was calculated. The image was visualized using Nikon ECLIPSE Ti (Fukasawa, Japan) and analyzed with Nikon software.

### Dual-luciferase reporter gene assay

The target sites of EGR1 and miR-130a were determined by bioinformatics websites miRanda, starBase and DIANA. EGR1 3’untranslated region (UTR)-wild-type (WT) and EGR1 3’UTR-mutant type (MUT) fragments were composed by Sangon with Not and Xho endonuclease cleavage sites at both ends, then recombined into the psi-CHECK2 polyclone sites. The WT and MUT sequences were identified. HCCLM3 cells were seeded into a 24-well plate. With 80% confluence, cells were transfected. The transfection reagent was arranged according to Lipofectamine^TM^ 2000 specification(Invitrogen Inc., Carlsbad, CA, USA). The dual-luciferase reporter vectors (50 ng) and miR-130a mimic or mimic NC (50 nmol/L) were co-transfected into HCCLM3 cells. The luciferase activity was verified by Dual-luciferase Report Assay System (Promega, Madison, WI, USA) 48 h later.

### Trypan blue staining

HCCLM3 cells which had been treated with 1, 10, 100 nmol/L DEX for 24 h were collected. HCCLM3 single-cell suspension (100 μL) and 100 μL trypan blue staining solution (Sigma–Aldrich, CA, USA) were reacted for 3 min. The dead cells were counted and recorded by a CountStar cell counter, and the cell viability was calculated.

### Cell transfection

When the optimal concentration of DEX on cells was determined, HCCLM3 cells were treated with 10 nmol/L DEX for 24 h and transfected with miR-130a mimic, mimic NC, miR-130a inhibitor or inhibitor NC. At the same time, cells were also transfected with miR-130a mimic, mimic NC, miR-130a inhibitor, inhibitor NC, si-EGR1, si-NC, oe-EGR1 or oe-NC, respectively. MiR-130a mimic, inhibitor, and the corresponding NCs were compounded by Shanghai Sangon Biotechnology Co. Ltd. (Shanghai, China) while oe-EGR1, si-EGR1, and the corresponding NCs were constructed by Ribobio (Guangzhou, China). Lipofectamin 2000 reagent (Invitrogen Inc., Carlsbad, CA, USA) was adopted for cell transfection.

### 3-(4, 5-dimethylthiazol-2-yl)-2, 5-diphenyltetrazolium bromide (MTT) assay

HCCLM3 cells were seeded into a 96-well plate at 5000 cells per well and detected for cell proliferation by MTT method (Promega Corporation, Madison, USA). MTT solution (5 mg/mL, 20 μL) was added to cells for 4 -h incubation. Then, cells were reacted with dimethyl sulfoxide (200 μL) to dissolve formazan. The optical density (OD_490 nm_) value was measured.

### Colony formation assay

HCCLM3 cells were seeded in six-well plates at 1 × 10^2^ cells/well. Cultured for 2 weeks, cells were fixed with 4% PFA (Solarbio, Beijing, China) and dyed with crystal violet staining solution (Beyotime Biotechnology, Shanghai, China) for 25 min. Colonies were photographed and reckoned (Nikon, Tokyo, Japan).

### Transwell assay

HCCLM3 cells (1 × 10^5^ cells) were suspended in 200 μL serum-free DMEM and cultured on the upper layer of the transwell chamber (without matrigel, BD Biosciences, NJ, USA) with 40 μL/well. The lower layer was added with 500 μL DMEM/10% FBS/50 μg fibronectin. Cells were dyed with 0.1% crystal violet staining solution. Five fields of view were randomly selected to count cells.

Transwell invasion experiment: matrigel (500 μL, BD Biosciences) was appended in the upper layer in advance and then added with cell suspension. The rest of the steps were the same as the migration experiment.

### Scratch test

HCCLM3 cells (5 × 10^5^ cells) were cultured in a 35 mm dish, with two parallel wells in each group. After the cells completely adhered to the wall, a 1 mL pipette tip was utilized to scratch the cell monolayer (the scratch was smooth, complete and continuous under the microscope). Cells falling off were washed by PBS, and observed under the light microscope. The cell growth at the scratch was observed and recorded, and the migration distance was recorded after continuing to be cultured for 1 day.

### Flow cytometry

HCCLM3 cells (1 × 10^6^ cells) were fixed by pre-cooled 70% ethanol overnight. Rinsed with cold PBS, cells were combined with 100 μL 1 × binding buffer, added with 5 μL Annexin V-fluorescein isothiocyanate and 5 μL PI for 10 min. Cell apoptosis was tested by a flow cytometer within 1 h.

### Statistical analysis

All data analysis was conducted using SPSS 19.0 software (IBM, NY, USA). The experimental results were showed as the mean ± standard deviation. The *t*-test was performed for comparisons between two groups and one-way analysis of variance (ANOVA) was used for comparisons among multiple groups, followed by Tukey’s post-hoc test. Statistical significance was set at *p* < 0.05.

## Supplementary information

cddiscovery-author-contribution-form

aj-checklist

## Data Availability

The original contributions presented in the study are included in the article/Supplementary Material, further inquiries can be directed to the corresponding author.
